# On a two-dimensional model of generalized thermoelasticity with application

**DOI:** 10.1038/s41598-022-19656-w

**Published:** 2022-09-16

**Authors:** Ethar A. A. Ahmed, A. R. El-Dhaba, M. S. Abou-Dina, A. F. Ghaleb

**Affiliations:** 1 School of Engineering and Applied Sciences, Nile University, Giza, 12588 Egypt; 2 Department of Mathematics, Faculty of Science, Damanhour University, Damanhour, Egypt; 3 Department of Mathematics, Faculty of Science, Cairo University, Giza, 12613 Egypt

**Keywords:** Materials science, Mathematics and computing, Applied mathematics, Computational science, Software

## Abstract

A 2D first order linear system of partial differential equations of plane strain thermoelasticity within the frame of extended thermodynamics is presented and analyzed. The system is composed of the equations of classical thermoelasticity in which displacements are replaced with velocities, complemented with Cattaneo evolution equation for heat flux. For a particular choice of the characteristic quantities and for positive thermal conductivity, it is shown that this system may be cast in a form that is symmetric *t*-hyperbolic without further recurrence to entropy principle. While hyperbolicity means a finite speed of propagation of heat waves, it is known that symmetric hyperbolic systems have the desirable property of well-posedness of Cauchy problems. A study of the characteristics of this system is carried out, and an energy integral is derived, that can be used to prove uniqueness of solution under some boundary conditions. A numerical application for a finite slab is considered and the numerical results are plotted and discussed. In particular, the wave propagation nature of the solution is put in evidence.

## Introduction

The hyperbolic systems of partial differential equations have been a subject of permanent interest, whether in the theory for their unique properties, or for their appicability to the phenomenon of wave propagation in several branches of Science and Technology. Problems of generalized thermoelasticity leading to heat wave propagation have provided great opportunity to investigate solutions of hyperbolic systems of partial differential equations under various initial and boundary conditions. Ruggeri^1^ presented a survey on the relations between mathematical problems for quasi-linear hyperbolic systems and extended thermodynamics in continuum theories. It was shown that the system of balance laws can be symmetrized by use of an appropriate choice of the field variables. A purely thermal case was treated by Wilmański^2^, ch. 9] using an undetermined multipliers technique. Müller^3^ investigated the symmetric hyperbolic systems of partial differential equations in extended thermodynamics. Selivanov and Selivanova^4^ studied the computability properties of a system of symmetric hyperbolic equations by a difference scheme. Othman et al.^5^ investigated generalized thermoelastic diffusion in a homogeneous, isotropic elastic half-space based on the Green-Naghdi theory. Abbas and Zenkour^6^ used finite elements to investigate a two-dimensional problem under Green and Naghdi theory of thermoelasticity for a fiber-reinforcement anisotropic half-space subjected to a thermal boundary shock to assess the effects of initial stress and rotation. Cimmelli et al.^7^ presented a review of the modern mathematical methods in the generalized thermodynamics of continuous media. He et al.^8^ studied a two-dimensional generalized thermoelastic diffusion problem for a half-space. Mishra^9^ solved a 2D-problem of heat transfer in a thin plate based on single-phase-lagging heat conduction by superposition technique and Fourier series expansion. Ghaleb et al.^10^ presented a model of electro-thermoelasticity within the theory of generalized thermodynamics. Abbas and Marin^11^ studied a 2D-problem of generalized thermoelasticity for a half-space with surface laser heating. Rogolino et al.^12^ derived two generalized heat-transport hyperbolic equations and studied thermodynamical compatibility for both. Jou^13^ considered some fundamental aspects of non-equilibrium thermodynamics with a view on heat transport in nanosystems. Mahmoud et al.^14^ investigated nonlinear heat wave propagation in rigid thermal conductors. Alzahrani *et al.*^15^ studied a two-dimensional problem of a porous medium in extended thermodynamics using spectral method. Ahmed *et al.*^16–18^ investigated two-dimensional problems of piezo-thermoelasticity within the dual-phase-lag model for layered media and a quarter-space. Solutions were presented based on normal modes technique, as well as numerical solutions by finite differences. Concerning the solution of systems of hyperbolic partial differential equations, Bonet et al.^19^ proposed a novel computational framework for thermoelasticity based on the use of a system of first order conservation laws. An explicit stabilised Petrov-Galerkin framework was proposed for the numerical solution of thermoelastic problems. Numerical examples were presented.

It thus appears from the exposed literature that 2D systems of equations of thermoelasticity in extended thermodynamics have been tackled in few cases with the aim of finding solutions to particular boundary-value problems. Analysis of such systems to explore the behaviour of solutions and well-posedness of Cauchy problems has been done only rarely sofar. The present work is situated in this context. A first order 2D linear system of plane strain thermoelasticity for a transverse isotropic material is presented within the frame of extended thermodynamics. The equations are those of classical thermoelasticity, supplemented by Cattaneo evolution equation for the heat flux. This system, in which velocities replace mechanical displacements, is shown to be reducible to a symmetric *t*-hyperbolic form for a particular choice of the characteristic quantities and for positive heat conduction coefficient, without further reference to an entropy principle. This result is to be contrasted to that stated by Müller^3^, according to which the set of quasi-linear first order balance equations of extended thermodynamics may be written in symmetric hyperbolic form by a suitable choice of fields, provided the entropy principle is observed. It is known that symmetric hyperbolic systems behave well, in the sense of well-posedness of the Cauchy problem, i.e existence, uniqueness and stability of solutions with respect to boundary conditions. A study of the characteristics of the system is carried out, and an energy integral is derived, that can be used to prove uniqueness of solution under some convenient boundary conditions. It is thus shown that the proposed system of equations is valid for the description of heat wave propagation. A numerical experiment is considered for the rectangular slab under specified boundary conditions and zero initial conditions using COMSOL Multiphysics software. Numerical results are produced for a particular solution and the obtained quantities of practical interest are represented graphically and discussed.

## The linear system of equations

Let $u_x,u_y$ denote the mechanical displacement components, $v_x,v_y$- the corresponding velocity components, $\sigma _{xx}, \sigma _{yy}, \sigma _{xy}$- the identically non-vanishing stress components in the plane, $\theta$- the temperature measured from a reference temperature $\theta _0$ and $q_x, q_y$- the heat flux components. The linear equations of plane generalized thermoelasticity for a transverse isotropic material within the theory of extended thermodynamics read:

1. Equations of motion
  1 $$\begin{aligned}{}&\rho \frac{\partial v_x}{\partial t} - \frac{\partial \sigma _{xx}}{\partial x} - \frac{\partial \sigma _{xy}}{\partial y} = 0, \end{aligned}$$
  2 $$\begin{aligned}{}&\rho \frac{\partial v_y}{\partial t} - \frac{\partial \sigma _{xy}}{\partial x} - \frac{\partial \sigma _{yy}}{\partial y} = 0, \end{aligned}$$
2. Heat equation
  3 $$\begin{aligned} \theta _0 \gamma \left( \frac{\partial v_x}{\partial x} + \frac{\partial v_y}{\partial y} \right) + \rho C_e \frac{\partial \theta }{\partial t} + \frac{\partial q_x}{\partial x} + \frac{\partial q_y}{\partial y} = 0, \end{aligned}$$
3. Cattaneo–Vernotte relations. These evolution laws for the heat flux components replace the classical Fourier law for heat conduction. Divided throughout by thermal relaxation times $\tau _1$ and $\tau _2$ respectively, these relations read:
  4 $$\begin{aligned}{}&\rho \theta _0 \frac{\partial q_x}{\partial t} + n_{11} q_x + m_{11} \frac{\partial \theta }{\partial x} = 0, \end{aligned}$$
  5 $$\begin{aligned}{}&\rho \theta _0 \frac{\partial q_y}{\partial t} + n_{22} q_y + m_{22} \frac{\partial \theta }{\partial y} = 0, \end{aligned}$$
4. The generalized Hooke’s law differentiated w.r.t. time, thus allowing to eliminate the mechanical displacement components in favour of the velocities:
  6 $$\begin{aligned}{}&\frac{\partial \sigma _{xx}}{\partial t} - \left( \lambda + 2 \mu \right) \frac{\partial v_x}{\partial x} - \lambda \frac{\partial v_y}{\partial y} + \gamma \frac{\partial \theta }{\partial t} = 0, \end{aligned}$$
  7 $$\begin{aligned}{}&\frac{\partial \sigma _{yy}}{\partial t} - \lambda \frac{\partial v_x}{\partial x} - \left( \lambda + 2 \mu \right) \frac{\partial v_y}{\partial y} + \gamma \frac{\partial \theta }{\partial t} = 0, \end{aligned}$$
  8 $$\begin{aligned}{}&\frac{\partial \sigma _{xy}}{\partial t} - \mu \frac{\partial v_x}{\partial y} - \mu \frac{\partial v_y}{\partial x} = 0. \end{aligned}$$

The problem thus reduces to the solution of eight basic partial differential equations of the first order. These equations involve eight unknowns: two velocity components, three identically non vanishing stress components, temperature and two heat flux components. Having resolved this system of equations, one can then find the mechanical displacement components by quadrature from the relations:

9 $$\begin{aligned}{}&\frac{\partial u_x}{\partial t} - v_x = 0, \end{aligned}$$

10 $$\begin{aligned}{}&\frac{\partial u_y}{\partial t} - v_y = 0. \end{aligned}$$

Here, $\rho$ is the mass density, $\lambda , \mu$- Lamé coefficients, $\gamma$- the thermoelastic coefficient and $m_{11}, m_{22}$ are constants related to the coefficients of heat conduction. Young’s modulus will be denoted *E* and Poisson’s ratio $\sigma$.

It is not difficult to anticipate a time damping of the heat wave, due to the terms involving $n_{11}$ and $n_{22}$ in the Cattaneo evolution equations. In order to relate the coefficients $m_{11}, m_{22}, n_{11}$ and $n_{22}$ in Eqs. (4 and 5) to known physical quantities, it is sufficient to compare these two equations to the well-known standard forms of the evolution equations for the heat flux (C.f.^10,21^):

$$\begin{aligned} \tau _1 \frac{\partial q_x}{\partial t} + q_x + k_{11} \frac{\partial \theta }{\partial x}= & {} 0, \\ \tau _2 \frac{\partial q_y}{\partial t} + q_y + k_{22} \frac{\partial \theta }{\partial y}= & {} 0, \end{aligned}$$

where $\tau _1$, $\tau _2$ are the thermal relaxation times for the *x*- and *y*-directions respectively, and $k_{11}$, $k_{22}$ are the thermal conductivities in these two directions. Direct comparison of these last two equations with (4) and (5) reveals that

$$\begin{aligned} m_{11} = \frac{ \rho \theta _0 k_{11}}{\tau _1}, \quad m_{22} = \frac{ \rho \theta _0 k_{22}}{\tau _2}, \quad n_{11} = \frac{ \rho \theta _0}{\tau _1}, \quad n_{22} = \frac{ \rho \theta _0}{\tau _2}. \end{aligned}$$

The above governing equations need to be cast in a convenient form for later work. For this, Eqs. (6 and 7) will be replaced by their symmetrized forms:

11 $$\begin{aligned}{}&\frac{\partial \left( \alpha \sigma '_{xx} + \beta \sigma '_{yy} \right) }{\partial t} - \frac{\partial v_x}{\partial x} = 0, \end{aligned}$$

12 $$\begin{aligned}{}&\frac{\partial \left( \alpha \sigma '_{yy} + \beta \sigma '_{xx} \right) }{\partial t} - \frac{\partial v_y}{\partial y} =0, \end{aligned}$$

where

13 $$\begin{aligned} \sigma '_{xx}= & {} \sigma _{xx} + \gamma \theta , \qquad \sigma '_{yy} = \sigma _{yy} + \gamma \theta , \nonumber \\ \alpha= & {} \frac{\lambda + 2 \mu }{4 \mu (\lambda + \mu )} = \frac{1 - \nu ^2}{E}, \qquad \beta = - \frac{\mu }{4 \mu (\lambda + \mu )} = - \frac{(1 + \nu )(1 - 2 \nu )}{2E}. \end{aligned}$$

It may be easily verified that

14 $$\begin{aligned} \alpha ^2 - \ \beta ^2 = \frac{1}{4 \mu (\lambda + \mu )} = \frac{(1 + \nu )^2(1 - 2 \nu )}{E^2} > 0. \end{aligned}$$

Excluding Eqs. (9 and 10) which may be considered independently in a later stage as explained above, the system of eight basic equations is written in matrix form as:

15 $$\begin{aligned} A \frac{\partial U}{\partial t} + B \frac{\partial U}{\partial x} + C \frac{\partial U}{\partial y} = F. \end{aligned}$$

Matrices *A*, *B* and *C* have dimension $(8 \times 8)$ and are given as:

$$\begin{aligned} A= & {} \left( \begin{array}{cccccccc} \rho &{} 0 &{} 0 &{} 0 &{} 0 &{} 0 &{} 0 &{} 0 \\ 0 &{} \rho &{} 0 &{} 0 &{} 0 &{} 0 &{} 0 &{} 0 \\ 0 &{} 0 &{} \alpha &{} \beta &{} 0 &{} 0 &{} 0 &{} 0 \\ 0 &{} 0 &{} \beta &{} \alpha &{} 0 &{} 0 &{} 0 &{} 0 \\ 0 &{} 0 &{} 0 &{} 0 &{} 1 &{} 0 &{} 0 &{} 0 \\ 0 &{} 0 &{} 0 &{} 0 &{} 0 &{} \rho C_e &{} 0 &{} 0 \\ 0 &{} 0 &{} 0 &{} 0 &{} 0 &{} 0 &{} \rho \theta _0 &{} 0 \\ 0 &{} 0 &{} 0 &{} 0 &{} 0 &{} 0 &{} 0 &{} \rho \theta _0 \end{array} \right) , \, B = \left( \begin{array}{cccccccc} 0 &{} 0 &{} -1 &{} 0 &{} 0 &{} \gamma &{} 0 &{} 0 \\ 0 &{} 0 &{} 0 &{} 0 &{} -1 &{} 0 &{} 0 &{} 0 \\ -1 &{} 0 &{} 0 &{} 0 &{} 0 &{} 0 &{} 0 &{} 0 \\ 0 &{} 0 &{} 0 &{} 0 &{} 0 &{} 0 &{} 0 &{} 0 \\ 0 &{} -\mu &{} 0 &{} 0 &{} 0 &{} 0 &{} 0 &{} 0 \\ \theta _0 \gamma &{} 0 &{} 0 &{} 0 &{} 0 &{} 0 &{} 1 &{} 0 \\ 0 &{} 0 &{} 0 &{} 0 &{} 0 &{} m_{11} &{} 0 &{} 0 \\ 0 &{} 0 &{} 0 &{} 0 &{} 0 &{} 0 &{} 0 &{} 0 \end{array} \right) , \\ \\ C= & {} \left( \begin{array}{cccccccc} 0 &{} 0 &{} 0 &{} 0 &{} -1 &{} 0 &{} 0 &{} 0 \\ 0 &{} 0 &{} 0 &{} -1 &{} 0 &{} \gamma &{} 0 &{} 0 \\ 0 &{} 0 &{} 0 &{} 0 &{} 0 &{} 0 &{} 0 &{} 0 \\ 0 &{} -1 &{} 0 &{} 0 &{} 0 &{} 0 &{} 0 &{} 0 \\ -\mu &{} 0 &{} 0 &{} 0 &{} 0 &{} 0 &{} 0 &{} 0 \\ 0 &{} \theta _0 \gamma &{} 0 &{} 0 &{} 0 &{} 0 &{} 0 &{} 1 \\ 0 &{} 0 &{} 0 &{} 0 &{} 0 &{} 0 &{} 0 &{} 0 \\ 0 &{} 0 &{} 0 &{} 0 &{} 0 &{} m_{22} &{} 0 &{} 0 \end{array} \right) , \quad F = \left( \begin{array}{cccccccc} 0 \\ 0 \\ 0 \\ - n_{11} \\ - n_{22} \\ 0 \\ 0 \\ 0 \end{array} \right) , \quad U = \left( \begin{array}{cccccccc} v_x \\ v_y \\ \sigma '_{xx} \\ \sigma '_{yy} \\ \sigma _{xy} \\ \theta \\ q_x \\ q_y \end{array} \right) . \end{aligned}$$

The following definitions are taken from Godunov^20^, pp. 85-88]:

### Definition 1

*A surface S with equation* $\phi \left( x,y,t \right) = 0$ *on which*

16 $$\begin{aligned} \text {det} \left( \frac{\partial \phi }{\partial t} A + \frac{\partial \phi }{\partial x} B + \frac{\partial \phi }{\partial y} C \right) = 0, \end{aligned}$$

*or, equivalently,*

17 $$\begin{aligned} \text {det} \left( \tau A + \xi B + \eta C \right) = 0, \end{aligned}$$

*where* $\left( \tau , \xi , \eta \right)$ *denotes a vector normal to the surface S , is called a characteristic for the system of Eq. *(15).

### Definition 2

*The system of Eq.* (15) * is said to be t-hyperbolic if its characteristic equation has exactly 8 real and different roots* $\tau$ *for arbitrary real values of the parameters *($\xi , \eta )$.

In the case of multiple roots, the system still preserves all the main properties of hyperbolic systems.

### Definition 3

*The system of Eq. *(15) * is said to be symmetric, t-hyperbolic if the matrices A, B, C are symmetric and, moreover, matrix A is positive definite.*

It is well-known that for symmetric, t-hyperbolic systems one can deduce the so-called *energy integral*, which represents a powerful tool to prove a theorem on the uniqueness of solution.

## Reduction to symmetric t-hyperbolic form

In what follows, we investigate the possibility of diagonalizing the matrix $\tau A + \xi B + \eta C$ for all values of $(\xi , \eta )$ and $\tau > 0$, in order to simplify the considered system of equations and reveal its nature. As a first step, the basic equations will now be reformulated in dimensionless form. To this end, introduce the following set of dimensionless variables for length, time, temperature heat flux, velocity and stress:

$$\begin{aligned} {\tilde{x}} = \frac{x}{L_0}, \quad {\tilde{y}} = \frac{y}{L_0}, \quad {\tilde{t}} = \frac{t}{T_0}, \quad {\tilde{\theta }} = \frac{\theta }{\theta _0}, \quad \tilde{{{\mathbf {q}}}} = \frac{{{\mathbf {q}}}}{Q_0}, \quad \tilde{{{\mathbf {v}}}} = \frac{{{\mathbf {v}}}}{c}, \quad {\tilde{\sigma }}_{xx}' = \frac{\sigma '_{xx}}{\mu }, \cdots \end{aligned}$$

The characteristic quantities $\theta _0, L_0, T_0, Q_0, c$ are kept undetermined at this stage, but will be defined later on step by step so as to achieve some properties and simplifications. The two characteristic quantities $L_0$ and $T_0$ are related to each other by:

$$\begin{aligned} \frac{L_0}{T_0} = c. \end{aligned}$$

It may be noticed that the characteristic quantities $\theta _0$ and $Q_0$ are independent of each other according to the basic assumption of extended thermodynamics adopted in the present model (see^10^, for example). This fact provides some flexibility in attaining the required final form of the equations.

After removing the tilde, the system of eight basic equations in dimensionless form reads:

18 $$\begin{aligned}{}&\frac{\partial v_x}{\partial t} - \frac{c_T^2}{c^2} \frac{\partial \sigma '_{xx}}{\partial x} - \frac{c_T^2}{c^2} \frac{\partial \sigma _{xy}}{\partial y} + \gamma ' \frac{\partial \theta }{\partial x} = 0, \end{aligned}$$

19 $$\begin{aligned}{}&\frac{\partial v_y}{\partial t} - \frac{c_T^2}{c^2} \frac{\partial \sigma _{xy}}{\partial x} - \frac{c_T^2}{c^2} \frac{\partial \sigma '_{yy}}{\partial y} + \gamma ' \frac{\partial \theta }{\partial y} = 0, \end{aligned}$$

20 $$\begin{aligned}{}&\frac{\partial \theta }{\partial t} + \frac{\gamma }{\rho C_e} \left( \frac{\partial v_x}{\partial x} + \frac{\partial v_y}{\partial y} \right) + \frac{Q_0}{\rho c C_e \theta _0} \left( \frac{\partial q_x}{\partial x} + \frac{\partial q_y}{\partial y} \right) = 0, \end{aligned}$$

21 $$\begin{aligned}{}&\frac{\partial \left( \alpha ' \sigma '_{xx} + \beta ' \sigma '_{yy} \right) }{\partial t} - \frac{\partial v_x}{\partial x} = 0, \end{aligned}$$

22 $$\begin{aligned}{}&\frac{\partial \left( \alpha ' \sigma '_{yy} + \beta ' \sigma '_{xx} \right) }{\partial t} - \frac{\partial v_y}{\partial y} =0, \end{aligned}$$

23 $$\begin{aligned}{}&\frac{\partial \sigma _{xy}}{\partial t} - \frac{\partial v_x}{\partial y} - \frac{\partial v_y}{\partial x} = 0, \end{aligned}$$

24 $$\begin{aligned}{}&\frac{\partial q_x}{\partial t} + \frac{n_{11} T}{\rho \theta _0} q_x + \frac{m_{11}}{\rho c Q_0} \frac{\partial \theta }{\partial x} = 0, \end{aligned}$$

25 $$\begin{aligned}{}&\frac{\partial q_y}{\partial t} + \frac{n_{22} T}{\rho \theta _0} q_y + \frac{m_{22}}{\rho c Q_0} \frac{\partial \theta }{\partial y} = 0, \end{aligned}$$

where

$$\begin{aligned} \alpha ' = \alpha \mu , \qquad \beta ' = \beta \mu , \qquad \gamma ' = \frac{\gamma \theta _0}{\rho c^2}. \end{aligned}$$

The matrices of the system now read:

$$\begin{aligned} A= & {} \left( \begin{array}{cccccccc} 1 &{} 0 &{} 0 &{} 0 &{} 0 &{} 0 &{} 0 &{} 0 \\ 0 &{} 1 &{} 0 &{} 0 &{} 0 &{} 0 &{} 0 &{} 0 \\ 0 &{} 0 &{} \alpha ' &{} \beta ' &{} 0 &{} 0 &{} 0 &{} 0 \\ 0 &{} 0 &{} \beta ' &{} \alpha ' &{} 0 &{} 0 &{} 0 &{} 0 \\ 0 &{} 0 &{} 0 &{} 0 &{} 1 &{} 0 &{} 0 &{} 0 \\ 0 &{} 0 &{} 0 &{} 0 &{} 0 &{} 1 &{} 0 &{} 0 \\ 0 &{} 0 &{} 0 &{} 0 &{} 0 &{} 0 &{} 1 &{} 0 \\ 0 &{} 0 &{} 0 &{} 0 &{} 0 &{} 0 &{} 0 &{} 1 \end{array} \right) , \, \, B = \left( \begin{array}{cccccccc} 0 &{} 0 &{} - \frac{c_T^2}{c^2} &{} 0 &{} 0 &{} \gamma ' &{} 0 &{} 0 \\ 0 &{} 0 &{} 0 &{} 0 &{} - \frac{c_T^2}{c^2} &{} 0 &{} 0 &{} 0 \\ -1 &{} 0 &{} 0 &{} 0 &{} 0 &{} 0 &{} 0 &{} 0 \\ 0 &{} 0 &{} 0 &{} 0 &{} 0 &{} 0 &{} 0 &{} 0 \\ 0 &{} -1 &{} 0 &{} 0 &{} 0 &{} 0 &{} 0 &{} 0 \\ \frac{\gamma }{\rho C_e} &{} 0 &{} 0 &{} 0 &{} 0 &{} 0 &{} \frac{Q_0}{\rho c C_e \theta _0} &{} 0 \\ 0 &{} 0 &{} 0 &{} 0 &{} 0 &{} \frac{m_{11}}{\rho c Q_0} &{} 0 &{} 0 \\ 0 &{} 0 &{} 0 &{} 0 &{} 0 &{} 0 &{} 0 &{} 0 \end{array} \right) , \\ C= & {} \left( \begin{array}{cccccccc} 0 &{} 0 &{} 0 &{} 0 &{} - \frac{c_T^2}{c^2} &{} 0 &{} 0 &{} 0 \\ 0 &{} 0 &{} 0 &{} - \frac{c_T^2}{c^2} &{} 0 &{} \gamma ' &{} 0 &{} 0 \\ 0 &{} 0 &{} 0 &{} 0 &{} 0 &{} 0 &{} 0 &{} 0 \\ 0 &{} -1 &{} 0 &{} 0 &{} 0 &{} 0 &{} 0 &{} 0 \\ -1 &{} 0 &{} 0 &{} 0 &{} 0 &{} 0 &{} 0 &{} 0 \\ 0 &{} \frac{\gamma }{\rho C_e} &{} 0 &{} 0 &{} 0 &{} 0 &{} 0 &{} \frac{Q_0}{\rho c C_e \theta _0} \\ 0 &{} 0 &{} 0 &{} 0 &{} 0 &{} 0 &{} 0 &{} 0 \\ 0 &{} 0 &{} 0 &{} 0 &{} 0 &{} \frac{m_{11}}{\rho c Q_0} &{} 0 &{} 0 \end{array} \right) , \, \, F = \left( \begin{array}{cccccccc} 0 \\ 0 \\ 0 \\ - \frac{n_{11} T}{\rho \theta _0} \\ - \frac{n_{22} T}{\rho \theta _0} \\ 0 \\ 0 \\ 0 \end{array} \right) . \end{aligned}$$

Matrices *B* and *C* can be made symmetric by suitable choices of some parameters, taking in consideration that some characteristic quantities of the system are still kept arbitrary. First, let us choose the characteristic velocity to be equal to the speed of propagation of the purely elastic transverse wave in the linear approximation:

$$\begin{aligned} c = c_T = \sqrt{\frac{\mu }{\rho }}. \end{aligned}$$

Next, assume the material parameters are such that the following relation holds:

$$\begin{aligned} \gamma ' = \frac{\gamma }{\rho C_e}, \end{aligned}$$

which amounts to defining the reference temperature as:

$$\begin{aligned} \theta _0 = \frac{\rho c^2}{\rho C_e} = \frac{\mu }{\rho C_e}. \end{aligned}$$

Finally, define the characteristic heat flux so that:

$$\begin{aligned} \frac{Q_0}{\rho c C_e \theta _0} = \frac{m}{\rho c Q_0} = M, \text {say} \rightarrow Q_0 = \sqrt{m C_e \theta _0}, \qquad m = m_{11} = m_{22}. \end{aligned}$$

The characteristic time $T_0$ will be determined depending on the thermal relaxation time in the numerical example treated below.

The final form of the basic system of eight equations is:

26 $$\begin{aligned}{}&\frac{\partial v_x}{\partial t} - \frac{\partial \sigma '_{xx}}{\partial x} - \frac{\partial \sigma _{xy}}{\partial y} + \gamma ' \frac{\partial \theta }{\partial x} = 0, \end{aligned}$$

27 $$\begin{aligned}{}&\frac{\partial v_y}{\partial t} - \frac{\partial \sigma _{xy}}{\partial x} - \frac{\partial \sigma '_{yy}}{\partial y} + \gamma ' \frac{\partial \theta }{\partial y} = 0, \end{aligned}$$

28 $$\begin{aligned}{}&\frac{\partial \theta }{\partial t} + \gamma ' \left( \frac{\partial v_x}{\partial x} + \frac{\partial v_y}{\partial y} \right) + M \left( \frac{\partial q_x}{\partial x} + \frac{\partial q_y}{\partial y} \right) = 0, \end{aligned}$$

29 $$\begin{aligned}{}&\frac{\partial \left( \alpha ' \sigma '_{xx} + \beta ' \sigma '_{yy} \right) }{\partial t} - \frac{\partial v_x}{\partial x} = 0, \end{aligned}$$

30 $$\begin{aligned}{}&\frac{\partial \left( \alpha ' \sigma '_{yy} + \beta ' \sigma '_{xx} \right) }{\partial t} - \frac{\partial v_y}{\partial y} =0, \end{aligned}$$

31 $$\begin{aligned}{}&\frac{\partial \sigma _{xy}}{\partial t} - \frac{\partial v_x}{\partial y} - \frac{\partial v_y}{\partial x} = 0, \end{aligned}$$

32 $$\begin{aligned}{}&\frac{\partial q_x}{\partial t} + n q_x + M \frac{\partial \theta }{\partial x} = 0, \end{aligned}$$

33 $$\begin{aligned}{}&\frac{\partial q_y}{\partial t} + n q_y + M \frac{\partial \theta }{\partial y} = 0, \end{aligned}$$

where

$$\begin{aligned} n = \frac{n_{11} T_0}{\rho \theta _0} = \frac{n_{22} T_0}{\rho \theta _0} \end{aligned}$$

for the transversely isotropic case under consideration.

By elimination, one easily deduces from the above equations the following expression for the dimensionless speed of the heat wave:

34 $$\begin{aligned} M = \frac{m}{\rho c Q_0} = \sqrt{\frac{m}{\rho \mu C_e \theta _0}} = \sqrt{\frac{k}{\tau \mu C_e}}. \end{aligned}$$

## Characteristics

In order to find the characteristics of the system of partial differential equations under consideration, note that the symmetric matrices *A*, *B* and *C* are now given by:

$$\begin{aligned} A= & {} \left( \begin{array}{cccccccc} 1 &{} 0 &{} 0 &{} 0 &{} 0 &{} 0 &{} 0 &{} 0 \\ 0 &{} 1 &{} 0 &{} 0 &{} 0 &{} 0 &{} 0 &{} 0 \\ 0 &{} 0 &{} \alpha ' &{} \beta ' &{} 0 &{} 0 &{} 0 &{} 0 \\ 0 &{} 0 &{} \beta ' &{} \alpha ' &{} 0 &{} 0 &{} 0 &{} 0 \\ 0 &{} 0 &{} 0 &{} 0 &{} 1 &{} 0 &{} 0 &{} 0 \\ 0 &{} 0 &{} 0 &{} 0 &{} 0 &{} 1 &{} 0 &{} 0 \\ 0 &{} 0 &{} 0 &{} 0 &{} 0 &{} 0 &{} 1 &{} 0 \\ 0 &{} 0 &{} 0 &{} 0 &{} 0 &{} 0 &{} 0 &{} 1 \end{array} \right) , \, \, B = \left( \begin{array}{cccccccc} 0 &{} 0 &{} -1 &{} 0 &{} 0 &{} \gamma ' &{} 0 &{} 0 \\ 0 &{} 0 &{} 0 &{} 0 &{} -1 &{} 0 &{} 0 &{} 0 \\ -1 &{} 0 &{} 0 &{} 0 &{} 0 &{} 0 &{} 0 &{} 0 \\ 0 &{} 0 &{} 0 &{} 0 &{} 0 &{} 0 &{} 0 &{} 0 \\ 0 &{} -1 &{} 0 &{} 0 &{} 0 &{} 0 &{} 0 &{} 0 \\ \gamma ' &{} 0 &{} 0 &{} 0 &{} 0 &{} 0 &{} M &{} 0 \\ 0 &{} 0 &{} 0 &{} 0 &{} 0 &{} M &{} 0 &{} 0 \\ 0 &{} 0 &{} 0 &{} 0 &{} 0 &{} 0 &{} 0 &{} 0 \end{array} \right) , \\ \\ C= & {} \left( \begin{array}{cccccccc} 0 &{} 0 &{} 0 &{} 0 &{} -1 &{} 0 &{} 0 &{} 0 \\ 0 &{} 0 &{} 0 &{} -1 &{} 0 &{} \gamma ' &{} 0 &{} 0 \\ 0 &{} 0 &{} 0 &{} 0 &{} 0 &{} 0 &{} 0 &{} 0 \\ 0 &{} -1 &{} 0 &{} 0 &{} 0 &{} 0 &{} 0 &{} 0 \\ -1 &{} 0 &{} 0 &{} 0 &{} 0 &{} 0 &{} 0 &{} 0 \\ 0 &{} \gamma ' &{} 0 &{} 0 &{} 0 &{} 0 &{} 0 &{} M \\ 0 &{} 0 &{} 0 &{} 0 &{} 0 &{} 0 &{} 0 &{} 0 \\ 0 &{} 0 &{} 0 &{} 0 &{} 0 &{} M &{} 0 &{} 0 \end{array} \right) , \quad F = \left( \begin{array}{cccccccc} 0 \\ 0 \\ 0 \\ - \frac{1}{\sqrt{2}} n \\ - \frac{1}{\sqrt{2}} n \\ 0 \\ 0 \\ 0 \end{array} \right) , \quad U = \left( \begin{array}{cccccccc} v_x \\ v_y \\ \sigma '_{xx} \\ \sigma '_{yy} \\ \sigma _{xy} \\ \theta \\ q_x \\ q_y \end{array} \right) \end{aligned}$$

and

$$\begin{aligned} det A = \left( \alpha '^2 - \beta '^2 \right) > 0. \end{aligned}$$

Matrix *A* is positive definite as can be directly verified, with eigenvalues: $1 ,\alpha ' +\beta ' ,\alpha ' -\beta '$, the first eigenvalue being repeated six times. The corresponding normalized eigenvectors are shown below together with their corresponding eigenvalues:

$$\begin{aligned} \left\{ \begin{array}{c} 1 \\ 0 \\ 0 \\ 0 \\ 0 \\ 0 \\ 0 \\ 0 \end{array} , \begin{array}{c} 0 \\ 1 \\ 0 \\ 0 \\ 0 \\ 0 \\ 0 \\ 0 \end{array} \right\} \leftrightarrow 1, \quad \left\{ \begin{array}{c} 0 \\ 0 \\ \frac{1}{\sqrt{2}} \\ \frac{1}{\sqrt{2}} \\ 0 \\ 0 \\ 0 \\ 0 \end{array} \right\} \leftrightarrow \alpha ' +\beta ', \quad \left\{ \begin{array}{c} 0 \\ 0 \\ -\frac{1}{\sqrt{2}} \\ \frac{1}{\sqrt{2}} \\ 0 \\ 0 \\ 0 \\ 0 \end{array} \right\} \leftrightarrow \alpha ' -\beta ', \quad \left\{ \begin{array}{c} 0 \\ 0 \\ 0 \\ 0 \\ 1 \\ 0 \\ 0 \\ 0 \end{array} , \begin{array}{c} 0 \\ 0 \\ 0 \\ 0 \\ 0 \\ 1 \\ 0 \\ 0 \end{array} , \begin{array}{c} 0 \\ 0 \\ 0 \\ 0 \\ 0 \\ 0 \\ 1 \\ 0 \end{array} , \begin{array}{c} 0 \\ 0 \\ 0 \\ 0 \\ 0 \\ 0 \\ 0 \\ 1 \end{array} \right\} \leftrightarrow 1. \end{aligned}$$

Introduce the matrix *T* whose columns are the eigenvectors of matrix *A*, arranged as shown above:

$$\begin{aligned} T = \left( \begin{array}{cccccccc} 1 &{} 0 &{} 0 &{} 0 &{} 0 &{} 0 &{} 0 &{} 0 \\ 0 &{} 1 &{} 0 &{} 0 &{} 0 &{} 0 &{} 0 &{} 0 \\ 0 &{} 0 &{} \frac{1}{\sqrt{2}} &{} -\frac{1}{\sqrt{2}} &{} 0 &{} 0 &{} 0 &{} 0 \\ 0 &{} 0 &{} \frac{1}{\sqrt{2}} &{} \frac{1}{\sqrt{2}} &{} 0 &{} 0 &{} 0 &{} 0 \\ 0 &{} 0 &{} 0 &{} 0 &{} 1 &{} 0 &{} 0 &{} 0 \\ 0 &{} 0 &{} 0 &{} 0 &{} 0 &{} 1 &{} 0 &{} 0 \\ 0 &{} 0 &{} 0 &{} 0 &{} 0 &{} 0 &{} 1 &{} 0 \\ 0 &{} 0 &{} 0 &{} 0 &{} 0 &{} 0 &{} 0 &{} 1 \end{array} \right) \end{aligned}$$

The similarity transformation with matrix *T*: $X' = T^T X T$ applied to matrices *A*, *B* and *C* yields the following:

$$\begin{aligned} A'= & {} \left( \begin{array}{cccccccc} 1 &{} 0 &{} 0 &{} 0 &{} 0 &{} 0 &{} 0 &{} 0 \\ 0 &{} 1 &{} 0 &{} 0 &{} 0 &{} 0 &{} 0 &{} 0 \\ 0 &{} 0 &{} \alpha ' +\beta ' &{} 0 &{} 0 &{} 0 &{} 0 &{} 0 \\ 0 &{} 0 &{} 0 &{} \alpha ' -\beta ' &{} 0 &{} 0 &{} 0 &{} 0 \\ 0 &{} 0 &{} 0 &{} 0 &{} 1 &{} 0 &{} 0 &{} 0 \\ 0 &{} 0 &{} 0 &{} 0 &{} 0 &{} 1 &{} 0 &{} 0 \\ 0 &{} 0 &{} 0 &{} 0 &{} 0 &{} 0 &{} 1 &{} 0 \\ 0 &{} 0 &{} 0 &{} 0 &{} 0 &{} 0 &{} 0 &{} 1 \end{array} \right) , \, B' = \left( \begin{array}{cccccccc} 0 &{} 0 &{} -\frac{1}{\sqrt{2}} &{} \frac{1}{\sqrt{2}} &{} 0 &{} \gamma ' &{} 0 &{} 0 \\ 0 &{} 0 &{} 0 &{} 0 &{} -1 &{} 0 &{} 0 &{} 0 \\ -\frac{1}{\sqrt{2}} &{} 0 &{} 0 &{} 0 &{} 0 &{} 0 &{} 0 &{} 0 \\ \frac{1}{\sqrt{2}} &{} 0 &{} 0 &{} 0 &{} 0 &{} 0 &{} 0 &{} 0 \\ 0 &{} -1 &{} 0 &{} 0 &{} 0 &{} 0 &{} 0 &{} 0 \\ \gamma ' &{} 0 &{} 0 &{} 0 &{} 0 &{} 0 &{} M &{} 0 \\ 0 &{} 0 &{} 0 &{} 0 &{} 0 &{} M &{} 0 &{} 0 \\ 0 &{} 0 &{} 0 &{} 0 &{} 0 &{} 0 &{} 0 &{} 0 \end{array} \right) , \\ C'= & {} \left( \begin{array}{cccccccc} 0 &{} 0 &{} 0 &{} 0 &{} -1 &{} 0 &{} 0 &{} 0 \\ 0 &{} 0 &{} -\frac{1}{\sqrt{2}} &{} -\frac{1}{\sqrt{2}} &{} 0 &{} \gamma ' &{} 0 &{} 0 \\ 0 &{} -\frac{1}{\sqrt{2}} &{} 0 &{} 0 &{} 0 &{} 0 &{} 0 &{} 0 \\ 0 &{} -\frac{1}{\sqrt{2}} &{} 0 &{} 0 &{} 0 &{} 0 &{} 0 &{} 0 \\ -1 &{} 0 &{} 0 &{} 0 &{} 0 &{} 0 &{} 0 &{} 0 \\ 0 &{} \gamma ' &{} 0 &{} 0 &{} 0 &{} 0 &{} 0 &{} M \\ 0 &{} 0 &{} 0 &{} 0 &{} 0 &{} 0 &{} 0 &{} 0 \\ 0 &{} 0 &{} 0 &{} 0 &{} 0 &{} M &{} 0 &{} 0 \end{array} \right) , \quad F' = \left( \begin{array}{cccccccc} 0 \\ 0 \\ - \frac{1}{2} n \\ - \frac{1}{2} n \\ - \frac{1}{\sqrt{2}} \frac{n}{\rho } \\ 0 \\ 0 \\ 0 \end{array} \right) , \quad U' = \left( \begin{array}{cccccccc} v_x \\ v_y \\ \frac{1}{\sqrt{2}} \left( \sigma '_{yy} + \sigma '_{xx} \right) \\ \frac{1}{\sqrt{2}} \left( \sigma '_{yy} - \sigma '_{xx} \right) \\ \sigma _{xy} \\ \theta \\ q_x \\ q_y \end{array} \right) . \end{aligned}$$

The new matrices are all symmetric. Moreover, $A'$ is positive definite. Thus, the transformed system of equations is symmetric, t-hyperbolic.

Further, consider the symmetric matrix $W = A'^{-1/2} \left( \xi B' + \eta C' \right) A'^{-1/2}$:

$$\begin{aligned} W = \left( \begin{array}{cccccccc} 0 &{} 0 &{} -\frac{1}{\sqrt{2}}\frac{\xi }{\sqrt{\alpha ' +\beta ' }} &{} \frac{1}{\sqrt{2}}\frac{\xi }{\sqrt{\alpha ' -\beta ' }} &{} - \eta &{} \gamma ' \xi &{} 0 &{} 0 \\ 0 &{} 0 &{} -\frac{1}{\sqrt{2}}\frac{\eta }{\sqrt{\alpha ' +\beta ' }} &{} -\frac{1}{\sqrt{2}}\frac{\eta }{\sqrt{\alpha ' -\beta ' }} &{} - \xi &{} \gamma ' \eta &{} 0 &{} 0 \\ -\frac{1}{\sqrt{2}}\frac{\xi }{\sqrt{\alpha ' +\beta ' }} &{} -\frac{1 }{\sqrt{2}}\frac{\eta }{\sqrt{\alpha ' +\beta ' }} &{} 0 &{} 0 &{} 0 &{} 0 &{} 0 &{} 0 \\ \frac{1}{\sqrt{2}}\frac{\xi }{\sqrt{\alpha ' -\beta ' }} &{} -\frac{1}{ \sqrt{2}}\frac{\eta }{\sqrt{\alpha ' -\beta ' }} &{} 0 &{} 0 &{} 0 &{} 0 &{} 0 &{} 0 \\ - \eta &{} - \xi &{} 0 &{} 0 &{} 0 &{} 0 &{} 0 &{} 0 \\ \gamma ' \xi &{} \gamma ' \eta &{} 0 &{} 0 &{} 0 &{} 0 &{} M\xi &{} M\eta \\ 0 &{} 0 &{} 0 &{} 0 &{} 0 &{} M\xi &{} 0 &{} 0 \\ 0 &{} 0 &{} 0 &{} 0 &{} 0 &{} M\eta &{} 0 &{} 0 \end{array} \right) . \end{aligned}$$

This matrix is diagonalized by means of a matrix *O*, say:

$$\begin{aligned} O^T \left( A'^{-1/2} \left( \xi B' + \eta C' \right) A'^{-1/2} \right) O = D. \end{aligned}$$

Then the two symmetric matrices $A'$ and $\left( \xi B' + \eta C' \right)$ are simultaneously diagonalizable by means of the transformation $S = A'^{-1/2}O$ for all real values of $(\xi , \eta )$. Moreover, the diagonal form of the first matrix is the unit matrix:

$$\begin{aligned} S^T A' S= & {} \left( O^T A'^{-1/2} \right) A' \left( A'^{-1/2}O \right) = O^T O = I, \\ S^T \left( \xi B' + \eta C' \right) S= & {} \left( O^T A'^{-1/2} \right) \left( \xi B' + \eta C' \right) \left( A'^{-1/2}O \right) = O^T W O = D. \end{aligned}$$

The diagonal matrix *D* may be obtained from the eigenvalues of matrix W. The characteristic equation of this matrix is:

35 $$\begin{aligned} \lambda ^2 \left( \lambda ^6 - \alpha _1 \lambda ^4 + \alpha _2 \lambda ^2 - \alpha _3 \right) = 0. \end{aligned}$$

The expressions for the coefficients are as follows:

$$\begin{aligned} \alpha _1= & {} \left( \xi ^2 + \eta ^2 \right) \left( 1 + \gamma '^2 + M^2 + \frac{\alpha '}{\alpha '^2 - \beta '^2} \right) , \\ \alpha _2= & {} \left[ \frac{1}{2} \frac{1}{\alpha ' - \beta '} + M^2 \left( 1 + \frac{\alpha '}{\alpha '^2 - \beta '^2} \right) \right] \left( \xi ^2 + \eta ^2 \right) ^2 \\&+ \left[ \frac{1}{2} \frac{1}{\alpha ' + \beta '} + \gamma '^2 \right] \left( \xi ^2 - \eta ^2 \right) ^2 + \frac{\left( 1 + \alpha ' + \beta ' \right) }{\alpha '^2 - \beta '^2} \, \xi ^2 \eta ^2, \\ \alpha _3= & {} \frac{M^2}{\alpha '^2 - \beta '^2} \left[ \alpha ' \left( \xi ^6 + \eta ^6 \right) + \xi ^2 \left( \xi ^2 + \eta ^2 \right) \left( 1 + \alpha ' + 2 \beta ' \right) \right] , \end{aligned}$$

from which it is seen that they are all positive. For sufficiently small absolute values of $\xi$ and $\eta$, the value of $\alpha _1$ is much larger than the values of the other two coefficients $\alpha _2$ and $\alpha _3$.

Consider the related cubic equation:

36 $$\begin{aligned} (f(z)=) z^3 - \alpha _1 z^2 + \alpha _2 z - \alpha _3 = 0. \end{aligned}$$

One easily verifies that

37 $$\begin{aligned} f'(0) = \alpha _2 > 0, \quad f''(0) = - 2 \alpha _1 <0. \end{aligned}$$

The local extrema of function *f* are located at:

38 $$\begin{aligned} z_1 = \alpha _1 - \sqrt{\alpha _1^2 - 3 \alpha _2}, \quad z_2 = \alpha _1 + \sqrt{\alpha _1^2 - 3 \alpha _2}. \end{aligned}$$

The discriminant for this cubic equation is:

39 $$\begin{aligned} \Delta = 18(\alpha _1)(\alpha _2)(\alpha _3 )-4(\alpha _1)^3(\alpha _3)+(\alpha _1)^2(\alpha _2)^2-4(\alpha _2)^3-27( \alpha _3)^2 \end{aligned}$$

**Figure 1:**
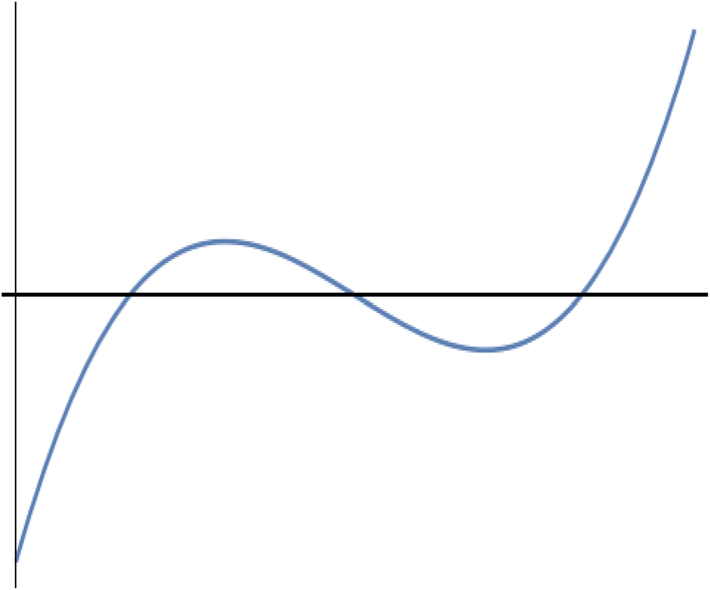
Cubic polynomial.

It is easy to see that sufficient conditions to get a curve shape for the cubic polynomial as in fig.1, i.e. for the eigenvalues to be real, are:

40 $$\begin{aligned} \alpha _1^2 - 3 \alpha _2 \ge 0, \quad \left( z_1^3 - \alpha _1 z_1^2 + \alpha _2 z_1 - \alpha _3 \right) \left( z_2^3 - \alpha _1 z_2^2 + \alpha _2 z_2 - \alpha _3 \right) \le 0. \end{aligned}$$

**Figure 2:**
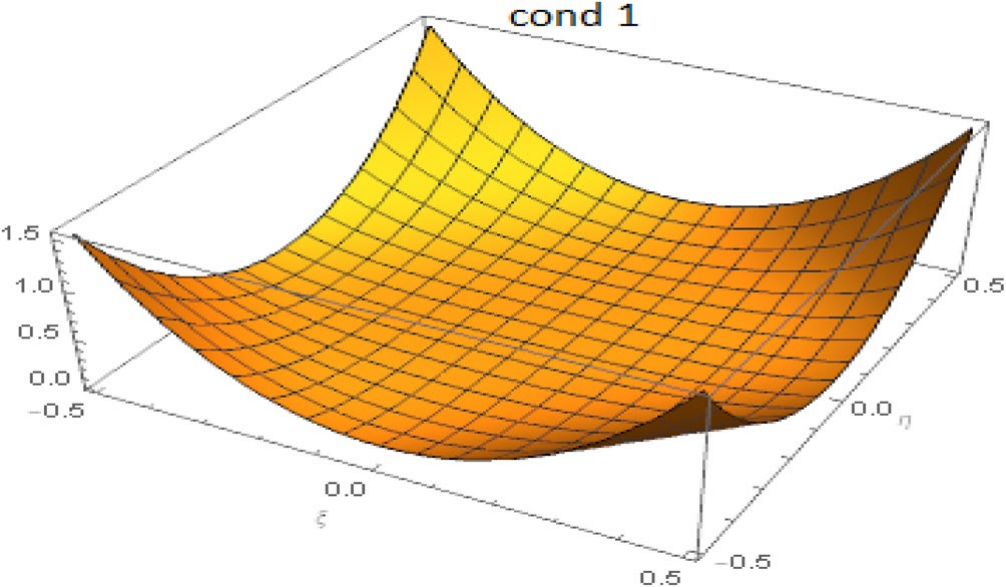
Satisfaction of the first condition for $-0.5 \le \xi , \eta \le 0.5$.

**Figure 3:**
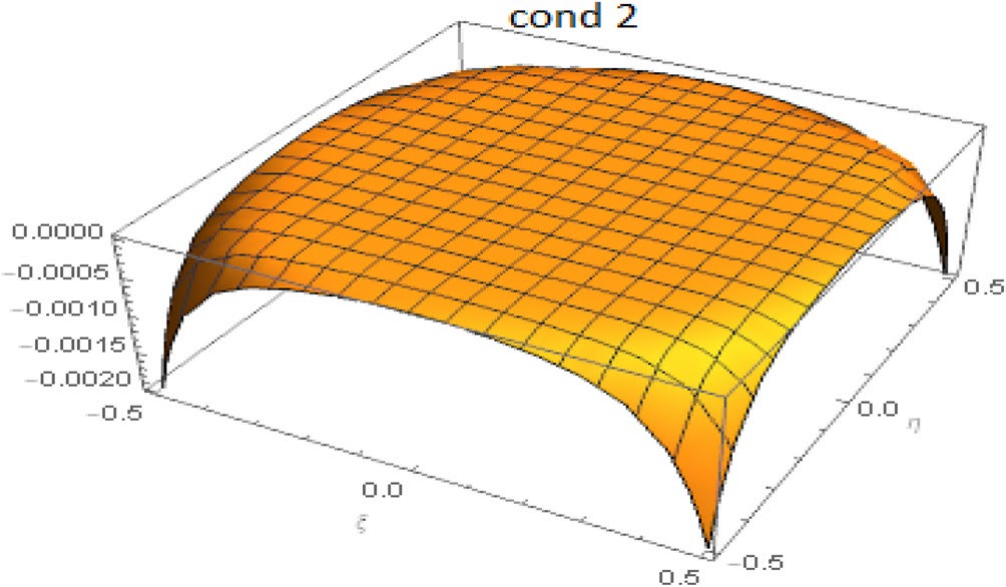
Satisfaction of the second condition for $-0.5 \le \xi , \eta \le 0.5$.

For the used values of the material parameters, these two conditions are likely to be satisfied for all values of $\xi$ and $\eta$. The fulfillment of the two conditions was achieved numerically as illustrated in Figs. 2 and 3. At the particular point $(\xi =0,\eta =0)$, all eigenvalues are coincident and equal to zero. Thus the Eq. (35) has eight real roots: A double root equal to zero, three positive roots, and three negative roots, the roots being symmetrically positioned with respect to the origin. One is finally led to the following:

### Theorem

*For all real values of the parameters *$\left( \xi , \eta \right)$*, the characteristic Eq.* (17) *of the considered linear system of equations has eight real roots symmetrically positioned w.r.t. origin of the real line: there are three positive roots, three negative roots and a zero root of multiplicity 2*.

## The energy integral

It is well-known (c.f.^20^) that an energy integral can be derived for symmetric t-hyperbolic systems of partial differential equations. If *V* denotes a region of the $\left( t, x, y \right)$-space included in the domain of definition of the solution, bounded by a surface *S*, then the energy integral for the transformed system reads^20^, p. 123]:

41 $$\begin{aligned} \iint _S \left( \left[ \tau A' + \xi B' + \eta C' \right] ,U' \right) \, ds = \iiint _V \left[ \left( D' U',U' \right) + 2 \left( F',U' \right) \right] \, dv, \end{aligned}$$

where operator $D'$ is defined as:

$$\begin{aligned} D' = \frac{\partial }{\partial t} A' + \frac{\partial }{\partial x} B' + \frac{\partial }{\partial y} C' \end{aligned}$$

and $\left( f,g \right)$ denotes the scalar product of the included functions. For the case under consideration, lengthy manipulations lead to the following form of the r.h.s. of (41):

42 $$\begin{aligned}{}&\iint _S \left( \left[ \tau A' + \xi B' + \eta C' \right] ,U' \right) \, ds \nonumber \\&\quad =\iint _S \tau \left( \vert {\varvec{v}} \vert ^2 + \vert {\varvec{q}} \vert ^2 + \theta ^2 + \left( \alpha ' -\beta ' \right) {\sigma '_{xx}}^2 + \left( \alpha ' + \beta ' \right) {\sigma '_{yy}}^2 + {\sigma '_{xy}}^2 \right) \, ds \nonumber \\&\qquad +2 \iint _S \left[ G \theta \, \varvec{v.} {\varvec{n}} + M \theta \, \varvec{q.} {\varvec{n}} - \left( \sigma '' {\varvec{v}} \right) \varvec{.n} \right] ds, \end{aligned}$$

where ${\varvec{n}} = \left( \xi , \eta \right)$ and we have introduced $\sigma ''$ with components:

$$\begin{aligned} \sigma ''_{xx} = \frac{1}{\sqrt{2}} \left( \sigma '_{yy} + \sigma '_{xx} \right) , \quad \sigma ''_{yy} = \frac{1}{\sqrt{2}} \left( \sigma '_{yy} - \sigma '_{xx} \right) , \quad \sigma ''_{xy} = \sigma '_{xy}. \end{aligned}$$

It is known that the characteristic surfaces for the wave equation are parts of conuses with axis parallel to the *t*-axis, so that $\tau > 0$. The boundary conditions are taken in the form:

43 $$\begin{aligned} \varvec{v.} {\varvec{n}} = \varvec{q.} {\varvec{n}} = \left( \sigma '' {\varvec{v}} \right) \varvec{.n} =0, \end{aligned}$$

yielding

44 $$\begin{aligned} \iiint _V \left[ \left( D' U',U' \right) + 2 \left( F',U' \right) \right] \, dv \ge 0. \end{aligned}$$

It is well-known that this last inequality leads to uniqueness of solution. The first two boundary conditions in (43) mean impermeability and thermal insulation of the boundary. The third one englobes many cases, among which complete fixing of the boundaries.

## Numerical application

As a confirmation of the existence of solutions to the investigated system of equations, we have considered a Cauchy problem for rectangular slab under specified boundary conditions. The solution includes the propagation of three types of waves, the heat wave, the transversal and the longitudinal thermomechanical coupled waves. Setting $\tau = \tau _1 = \tau _2$ the thermal relaxation time and $k= k_{11} = k_{22}$ the coefficient of heat conduction, one has to make assumptions concerning the orders of magnitude of constants appearing in (4) and (5):

$$\begin{aligned} n_{11}, n_{22} \sim \frac{\rho \theta _0}{\tau }, \quad m_{11}, m_{22} \sim \frac{k \rho \theta _0}{\tau }. \end{aligned}$$

Thus the dimensionless parameters *M* and *n* appearing in the dimensionless Cattaneo Eqs. (32 and 33) satisfy the rules

$$\begin{aligned} M \sim \sqrt{\frac{k}{\mu C_e \tau }}, \qquad n \sim \frac{T_0}{\tau } . \end{aligned}$$

For the example treated below, one has $M \sim 1$ and $n \sim 1$, hence

$$\begin{aligned} \tau \sim \frac{k}{\mu C_e}, \qquad T_0 \sim \tau . \end{aligned}$$

With this in mind, let us consider the case of a transversely isotropic material having tentative values of the different geometrical and material parameters shown in Table 1. Consultation of the available tables of material coefficients for metals and alloys reveals that the used values of mass density and specific heat capacity as displayed in Table 1 are within the normal range, while the thermal conductivity lies in the higher range.

**Table 1:** Values of the material parameters.

$a = 0.4~m$	$b = 0.4~m$	$\tau = 0.444 \times 10^{-10}~s$
$\theta _{0} = 300~K$	$\rho = 0.333 \times 10^{4}~kg/m^{3}$	$\nu =0.435$
$E =0.861 \times 10^{10}~kg/\left( m.s^{2} \right)$	$\lambda = 2.000 \times 10^{10}~kg/\left( m.s^{2} \right)$	$\mu =0.300 \times 10^{10}~kg/\left( m.s^{2}\right) \, \,$
$\gamma = 0.230 \times 10^{-4}~1/K$	$C_{e}= 0.300 \times 10^{4}~J/\left( kg.K\right)$	$k =400~W/\left( m.K\right)$

Corresponding to these values:

$$\begin{aligned} L_0 \sim 10^{-8} \, m, \qquad T_0 \sim 10^{-11} \, s, \qquad c \sim 10^3 \, m/s. \end{aligned}$$

**Figure 4:**
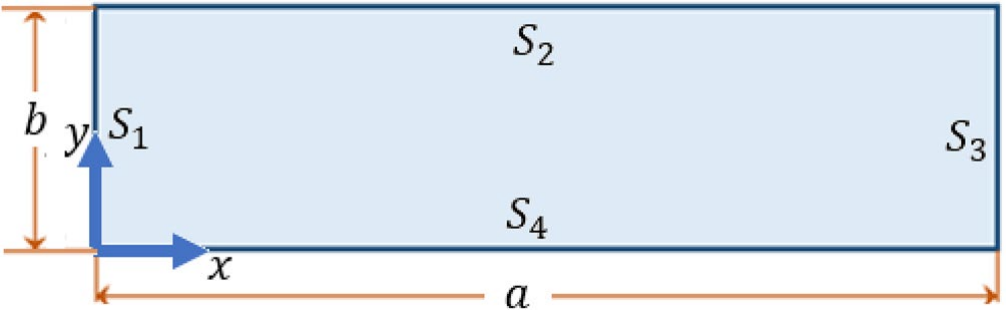
Schematic representation of the problem.

Motion is induced by a thermal boundary regime. For this, consider a rectangular domain occupied by a thermoelastic material, with dimensions *a* and *b* and sides labeled $S_1$, $S_2$, $S_3$ and $S_4$ as shown in Fig. 4. The origin of the plane Cartesian coordinate system is chosen at the left lower corner of the rectangle, with *x*-axis along the side $S_4$.

Initially, the medium is at rest at zero temperature (measured from an initial ambient temperature $\theta _0$) and heat flux, and in a stress-free state. The chosen boundary conditions are of mixed type. Mechanically, sides $S_1$, $S_2$ and $S_4$ are traction-free, side $S_3$ is completely fixed so that to suppress rigid body motion of the slab. As concerns the thermal conditions, the normal heat flux is taken to vanish on boundaries $S_2, S_3$ and $S_4$, together with Robin thermal condition. Side $S_1$ has a prescribed heating regime that generates the motion. It should be remembered that temperature and heat flux are independent thermodynamical quantities. For definiteness:

$(\mathtt{1})$ At the side $S_1 \, (x=0, 0 \le y \le b)$:

45 $$\begin{aligned} \sigma _{xx}^{S_1}\left( 0,y,t\right) =\sigma _{xy}^{S_1}\left( 0,y,t\right) =0 \end{aligned}$$

and

46 $$\theta ^{{S_{1} }} \left( {0,y,t} \right) = \left\{ {\begin{array}{*{20}l} {200y\left( {b - y} \right)t\exp \left( { - 0.1t} \right),} \hfill & {0 \le t \le 5 \times 10^{{ - 3}} } \hfill \\ {0,} \hfill & {t > 5 \times 10^{{ - 3}} } \hfill \\ \end{array} } \right.,\quad 0 \le y \le b$$

**Figure 5:**
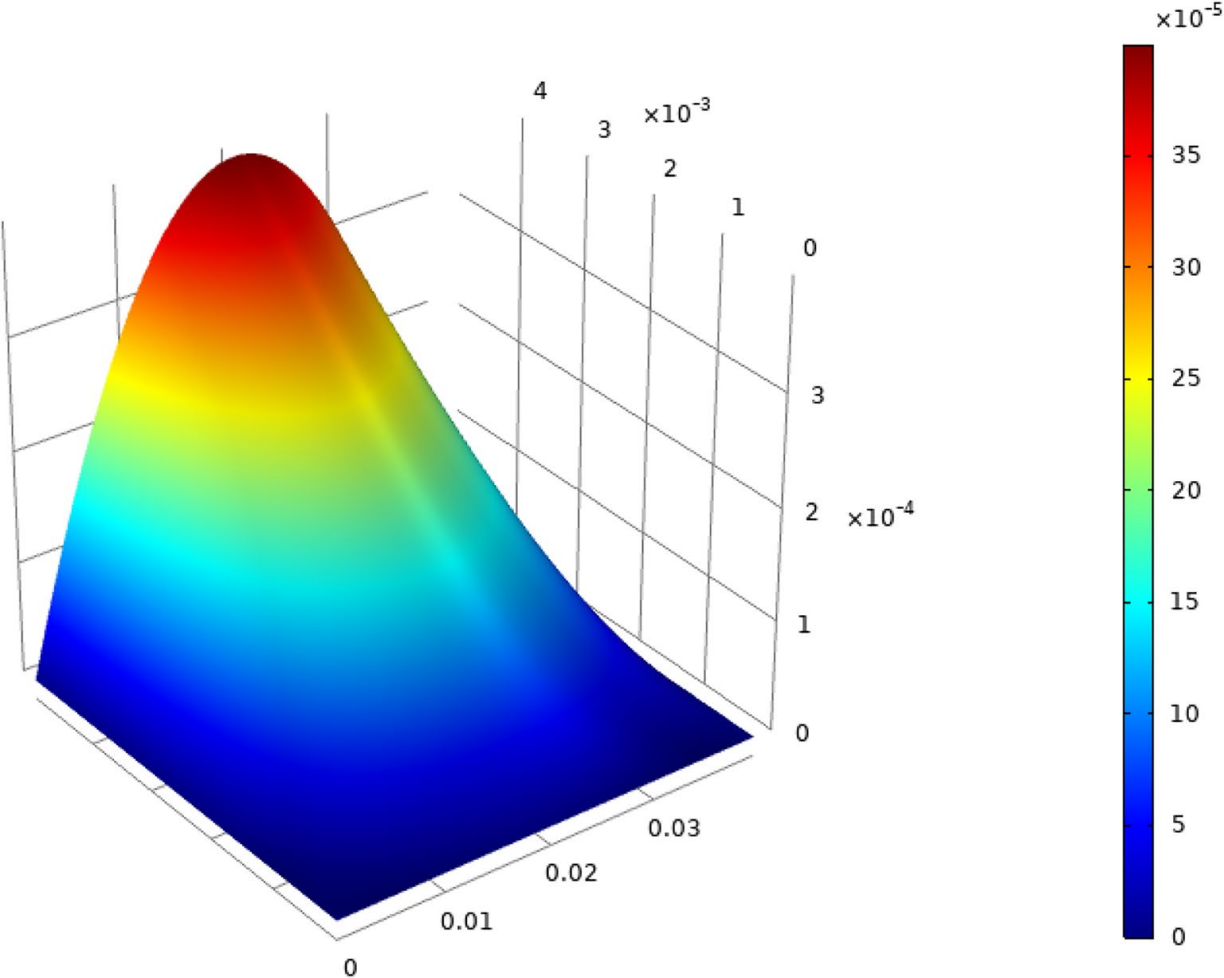
The applied thermal boundary condition.

The graphical representation of this function for $b=0.4$ is illustrated in Fig. 5.

$(\mathtt{2})$ At the side $S_2 \, (y=b, 0 \le x \le a)$:

47 $$\begin{aligned} \sigma _{yy}^{S_2}\left( x,b,t\right) =\sigma _{xy}^{S_2}\left( x,b,t\right) =q_{y}^{S_2}\left( x,b,t\right) =0, \end{aligned}$$

together with Robin thermal condition

48 $$\begin{aligned} \frac{\partial \theta ^{S_2}}{\partial y}\left( x,b,t\right) + Bi \, \, \theta ^{S_2} =0, \end{aligned}$$

$(\mathtt{3})$ At the side $S_3 \, (x=a, 0 \le y \le b)$:

49 $$\begin{aligned} \sigma _{xx}^{S_3}\left( a,y,t\right) =\sigma _{xy}^{S_3}\left( a,y,t\right) =q_{x}^{S_3}\left( a,y,t\right) =u_x \left( a,y,t \right) =u_y \left( a,y,t \right) = 0 \end{aligned}$$

and Robin thermal condition

50 $$\begin{aligned} \frac{\partial \theta ^{S_3}}{\partial x}\left( a,y,t\right) + Bi \, \, \theta ^{S_3} =0, \end{aligned}$$

$(\mathtt{4})$ At the side $S_4 \, (y=0, 0 \le x \le a)$:

51 $$\begin{aligned} \sigma _{yy}^{S_4}\left( x,0,t\right) =\sigma _{xy}^{S_4}\left( x,0,t\right) =q_{y}^{S_4}\left( x,0,t\right) =0 \end{aligned}$$

and Robin thermal condition

52 $$\begin{aligned} -\frac{\partial \theta ^{S_4}}{\partial y}\left( x,0,t\right) + Bi \, \theta ^{S_4} =0. \end{aligned}$$

Here, *Bi* is a dimensionless Biot number, with value taken as $Bi = 0.0144$. All the remaining functions not specified in the above boundary conditions are set to zero on the boundaries.

Following similar guidelines as in^22,23^, COMSOL Multiphysics is used to solve the considered boundary-value problem for Eqs. (26–33) under the boundary conditions (46–52) and zero initial conditions. For definiteness, we mainly consider the case with $M=3.0, n=1.0$. The value of *M*, however, was varied to take on other values in some plots. The produced particular solution has been obtained by the method of finite elements embedded in the above-mentioned software. The mesh could be refined and adjusted for best results.

From symmetry of the considered problem about the median line $y=b/2$, it follows that the quantities $u_y, v_y, q_y, \sigma _{xy}$ must vanish everywhere in the slab. The numerical computations have confirmed this fact . It thus follows that the displacements, velocities and heat flux take place along the direction perpendicular to the heated face of the slab (the *x*-direction), and that shear stress vanishes inside the slab.

**Figure 6:**
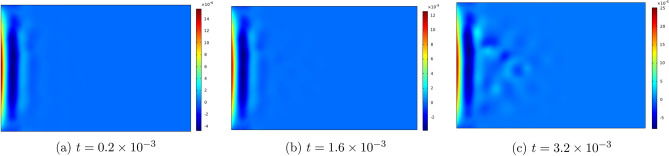
Top view of the temperature distribution in the slab at three consecutive time moments.

**Figure 7:**
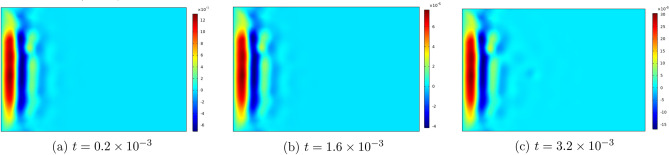
Top view of the distribution of $q_x$ in the slab at three consecutive time moments.

**Figure 8:**
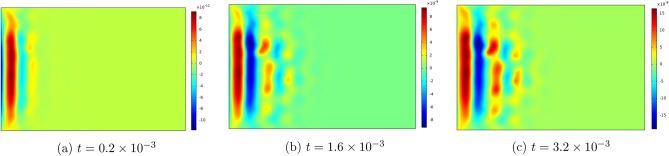
Top view of the distribution of $u_x$ in the slab at three consecutive time moments.

**Figure 9:**
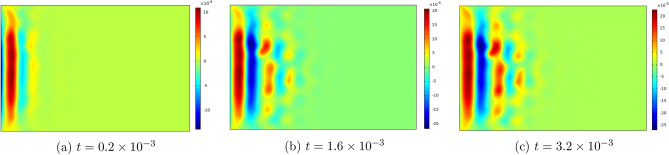
Top view of the distribution of $v_x$ in the slab at three consecutive time moments.

**Figure 10:**
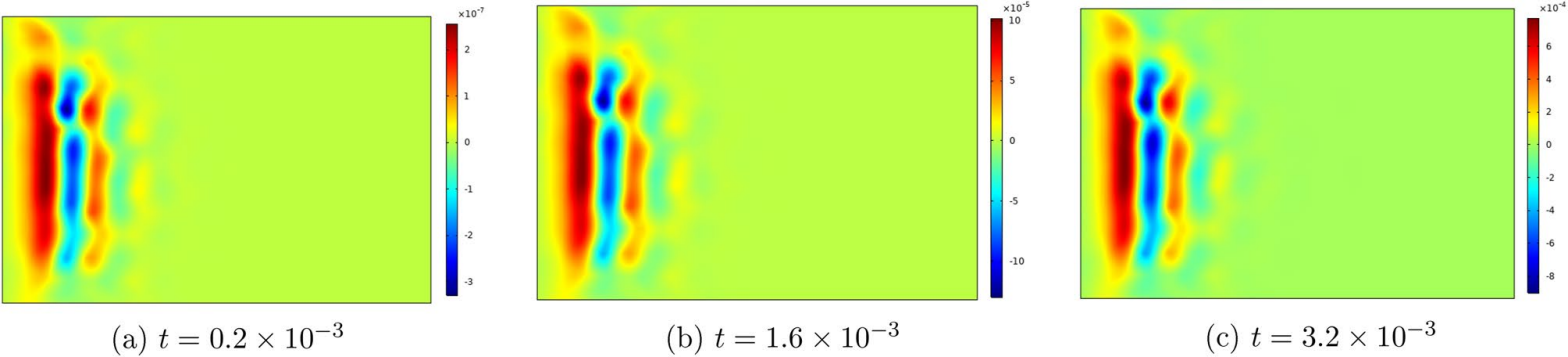
Top view of the distribution of $\sigma _{xx}$ in the slab at three consecutive time moments.

**Figure 11:**
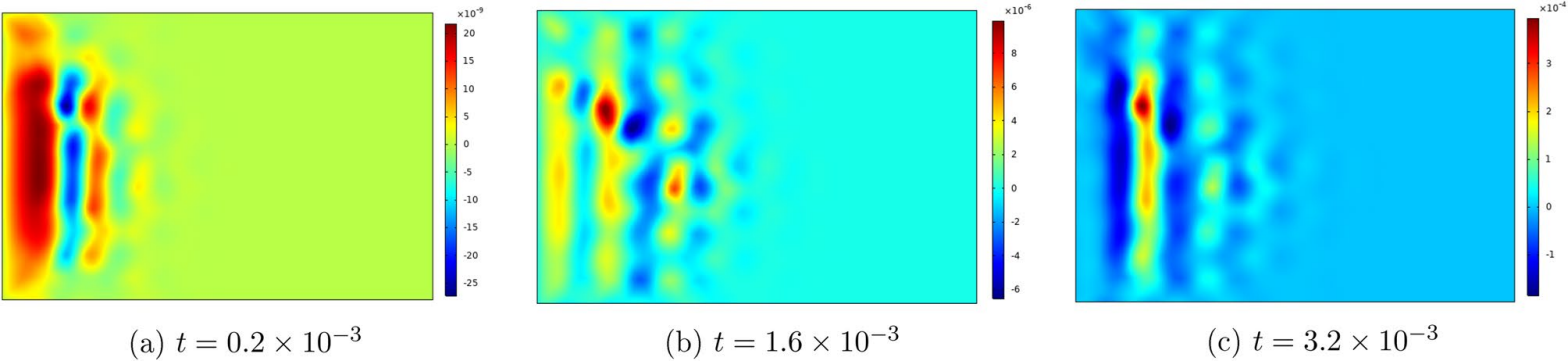
Top view of the distribution of $\sigma _{yy}$ in the slab at three consecutive time moments.

Figures 6, 7, 8, 9 , 10 and 11 represent topviews of the 3-D distributions at three consecutive time moments of the main physical quantities induced inside the material due to applied boundary conditions.

The calculations are performed for a dimensionless heat wave speed $M=3$. This wave thus travels three times faster than the transversal coupled thermoelastic wave whose dimensionless speed is $c=1.0$. For the chosen values of the material constants, the longitudinal coupled thermoelastic wave travels at speed $\simeq 2.944$. In all these figures, wave propagation phenomenon is clearly illustrated for both the mechanical and the thermal variables, with oscillations between positive and negative values near the heated side of the slab, and amplitudes monotonically decreasing to zero with distance. For the considered time values, the wave fronts are clearly noticed and the propagating disturbances have not yet reached the far end of the slab and therefore no reflected waves are expected. Larger time values could not be considered due to lack of stability of the computational scheme.

The first remark concerns the distribution of temperature in the slab. Although the motion was initiated by heating of the left boundary for a finite lapse of time, it is noticed in Fig. 6 that temperature assumes negative values near this boundary for the considered values of time. This seemingly paradoxical situation may be explained by the fact that part of the thermal energy supplied to the medium at the boundary is spent to generate mechanical wave propagation and, therefore, cooling may take place in some parts of the medium. Again, as shown by Ahmed et al.^24^ for the case of a rigid thermal conductor, negative temperatures can result from unphysical values assigned to the thermal relaxation time.

As noted above, heat flux and temperature are independent thermodynamical variables. Heat flux is initially generated by the thermal boundary condition. In subsequent time moments, it propagates as a wave, with speed *M*, independently of temperature as shown in Fig. 7.

Still at the heated end, the displacement and the velocity components $u_x$ and $v_x$ in Figs. 8 and  9 take on negative values in the initial phase of the motion, as the medium expands under heating. Both quantities are seen to tend to zero as the corner points are approached.

**Figure 12:**
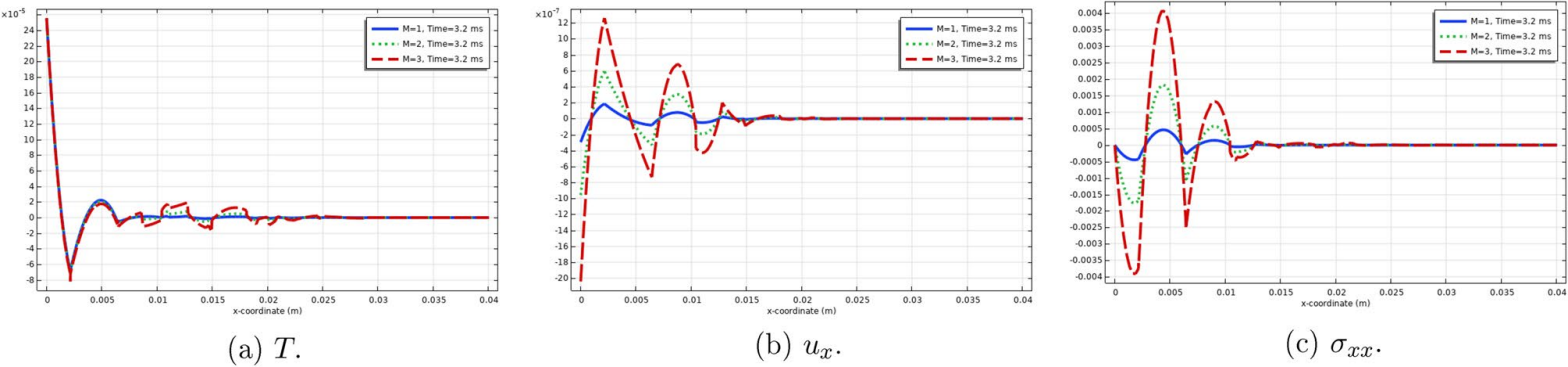
Distributions of $T, u_x$ and $\sigma _{xx}$ on the median line $y=b/2$ for $t=3.2 \times 10^{-3}$ and three values of *M*.

The stress components $\sigma _{xx}$ and $\sigma _{yy}$ are illustrated in Figs. 10 and  11. These two components take on negative values near the heated end of the slab, while $\sigma _{yy}$ assumes zero value at the ends $y=0,b$ in conformity with the applied boundary conditions. In order to confirm the satisfaction of the boundary condition for the first stress components, we have produced 2D-Fig. 12 for the distributions of the functions $\theta , u_x$ and $\sigma _{xx}$ on the median line $y=b/2$ for time value $t=3.2 \times 10^{-3}$ and for three values of parameter *M*, from which it is seen that the stress component $\sigma _{xx}$ in fact satisfies the boundary condition on $S_1$. These figures clearly show the effect of increase of parameter *M* on the amplitudes of the functions.

## Conclusions

We have investigated a two-dimensional system of first-order, partial differential equations of classical thermoelasticity, supplemented with Cattaneo-type evolution equation for the heat flux to replace Fourier law for heat conduction. The model includes only one thermal relaxation time for each component of the heat flux vector. It differs from other well-known models of extended thermodynamics, e.g. Lord and Shulman, Green and Naghdi, two-temperature model, dual-phase-lag model, which all start with different assumptions and yield heat wave propagation (C.f.^25^). Under the assumption that thermal conductivity must be positive, it turns out that the present system is reducible to symmetric t-hyperbolic form by special choices of some characteristic quantities. This result is to be contrasted with that expressed by Müller^3^, stating that the set of quasi-linear first order balance equations of extended thermodynamics may be written in symmetric hyperbolic form by a suitable choice of fields, provided constitutive functions are subject to the requirements of the entropy principle. The characteristics of the system were studied and an energy integral was obtained which leads to uniqueness of solution under proper boundary conditions. Thus the present system of equations behaves well in the sense of well-posedness of Cauchy problems, and is therefore valid for the description of heat wave propagation. To confirm the existence of solutions, a numerical experiment for a finite slab was carried out using a finite element scheme built in COMSOL Multiphysics and with tentative values of the different material parameters to produce a particular solution to the problem under specified boundary conditions and zero initial conditions. The numerical results could be obtained only for sufficiently small time values due to difficulties related to the stability of the numerical scheme. The presented three-dimensional plots clearly show the phenomenon of thermal and thermomechanical wave propagation. It was noticed that temperature attains negative values at certain locations inside the slab, although the left boundary is subjected to heating for a certain lapse of time. This is due to the fact that part of the supplied thermal energy is spent on generating the motion. Such phenomenon should not appear in rigid thermal conductors once the value of the thermal relaxation time has been properly assigned (C.f.^24^). The presented figures show the wave front and the damping of the solution as disturbances progress along the slab. Verification of the satisfaction of the boundary conditions was carried out.

## Data Availability

All data generated or analyzed during this study are included in this published article

## References

[1] Ruggeri, T. Thermodynamics and symmetric hyperbolic systems. *Rend. Sem. Mat. Univ. Pol. Torino, Fascicolo Speciale Hyperbolic equations* **1987**, 167–183 (1988).

[2] Wilmański K (1998). Thermomechanics of Continua.

[3] Müller I (2008). Extended thermodynamics: A theory of symmetric hyperbolic field equations. Entropy.

[4] Selivanova S, Selivanov V (2008). Computing solutions of symmetric hyperbolic systems of PDE’s. Electronic Notes Theoret. Comp. Sci..

[5] Othman MIA, Atwa SY, Farouk RM (2009). The effect of diffusion on two-dimensional problem of generalized thermoelasticity with Green-Naghdi theory. Int. Comm. Heat Mass Transf..

[6] Abbas IA, Zenkour AM (2014). The effect of rotation and initial stress on thermal shock problem for a fiber-reinforced anisotropic half-space using Green-Naghdi theory. J. Comput. Theoret. Nanosci..

[7] Cimmelli VA, Jou D, Ruggeri T, Ván P (2014). Entropy principle and recent results in non-equilibrium theories. Entropy.

[8] He T, Li C, Shi S, Ma Y (2015). A two-dimensional generalized thermoelastic diffusion problem for a half-space. Eur. J. Mech. A/Solids.

[9] Mishra TN (2015). Analytical solution of 2D SPL heat conduction model. Int. J. Latest Res. Engng. Techn. (IJLRET).

[10] Ghaleb AF, Abou-Dina MS, Rawy EK, El-Dhaba’ AR (2017). A model of nonlinear thermo-electroelasticity in extended thermodynamics. Int. J. Engng. Sci..

[11] Abbas IA, Marin M (2018). Analytical solutions of a two-dimensional generalized thermoelastic diffusions problem due to laser pulse. Iran. J. Sci. Technol. Trans. Mech. Eng..

[12] Rogolino P, Kovács R, Ván P, Cimmelli VA (2018). Generalized heat-transport equations: Parabolic and hyperbolic models. Contin. Mech. Thermodyn..

[13] Jou D (2020). Relationships between rational extended thermodynamics and extended irreversible thermodynamics. Phil. Trans. R. Soc. A.

[14] Mahmoud W, Moatimid GM, Abou-Dina MS, Ghaleb AF (2020). Nonlinear heat wave propagation in a rigid thermal conductor. Acta Mech..

[15] Alzahrani F, Hobiny A, Abbas I, Marin M (2020). An eigenvalues approach for a two-dimensional porous medium based upon weak, normal and strong thermal conductivities. Symmetry.

[16] Ahmed, Ethar A.A., Abou-Dina, M.S., Ghaleb, A.F. & Mahmoud, W. Numerical solution to a 2D problem of piezo-thermoelasticity in a quarter-space within the dual-phase-lag model. *Mater. Sci. Engng. B* **263**, 114790 (2021).

[17] Ahmed, Ethar A.A., Abou-Dina, M. S. & Ghaleb, A.F. Magnetic field effect on piezo-thermoelastic wave propagation in a half-space within dual-phase-lag. *Indian J. Phys.* **95**(6), 1101–1111. 10.1007/s12648-020-01779-3 (2021).

[18] Ahmed Ethar A.A., El-Dhaba AR, Abou-Dina MS, Ghaleb AF (2021). Thermoelastic wave propagation in a piezoelectric layered half-space within the dual-phase-lag model. Eur. Physics J. Plus.

[19] Bonet J, Leeb CH, Gil AJ, Ghavamian A (2021). A first order hyperbolic framework for large strain computational solid dynamics. Part III: Thermo-elasticity. Comput. Meth. Appl. Mech. Engrg..

[20] Godunov, C. K. *Equations of mathematical physics, *Nauka, Moscow, **(in Russian**)(1979).

[21] Liu Q, Peng Q, Ming P (2021). A control volume finite element method for the thermoelastic problem in functional graded material with one relaxation time. Proc. Institution Mech. Eng. Part C J. Mech. Eng. Sci..

[22] El-Dhaba AR, Mousavi SM (2021). Analysis of planes within reduced micromorphic model. Sci. Rep..

[23] El-Dhaba AR, Lim CW (2021). Dynamic response of composite materials with 2D reduced micromorphic model. Acta Mech. Solida Sin..

[24] Ahmed, Ethar A. A., Abou-Dina, M.S. & Ghaleb, A.F. Two-dimensional heat conduction in a rigid thermal conductor within the dual-phase-lag model by one-sided Fourier transform Waves Random Complex Media 10.1080/17455030.2020.1854492

[25] Youssef HM, El-Bary AA (2018). Theory of hyperbolic two-temperature generalized thermoelasticity. Mater. Phys. Mech..

